# Mapping the scientific output of organoids for animal and human modeling infectious diseases: a bibliometric assessment

**DOI:** 10.1186/s13567-024-01333-7

**Published:** 2024-06-26

**Authors:** Jin Yan, Jean Monlong, Céline Cougoule, Sonia Lacroix-Lamandé, Agnès Wiedemann

**Affiliations:** 1 Department of Gastroenterology, The Second Xiangya Hospital of Central South University, Changsha, China; 2 Research Center of Digestive Disease, Central South University, Changsha, China; 3 IRSD - Digestive Health Research Institute, University of Toulouse, INSERM, INRAE, ENVT, UPS, Toulouse, France; 4 Institut de Pharmacologie Et Biologie Structurale, IPBS, Université de Toulouse, CNRS, UPS, Toulouse, France; 5 ISP, INRAE, University of Tours, 37380 Nouzilly, France

**Keywords:** Organoid, pathogen, infection, Zika virus, SARS-CoV-2, bibliometric

## Abstract

The escalation of antibiotic resistance, pandemics, and nosocomial infections underscores the importance of research in both animal and human infectious diseases. Recent advancements in three-dimensional tissue cultures, or “organoids”, have revolutionized the development of in vitro models for infectious diseases. Our study conducts a bibliometric analysis on the use of organoids in modeling infectious diseases, offering an in-depth overview of this field’s current landscape. We examined scientific contributions from 2009 onward that focused on organoids in host‒pathogen interactions using the Web of Science Core Collection and OpenAlex database. Our analysis included temporal trends, reference aging, author, and institutional productivity, collaborative networks, citation metrics, keyword cluster dynamics, and disruptiveness of organoid models. VOSviewer, CiteSpace, and Python facilitated this analytical assessment. The findings reveal significant growth and advancements in organoid-based infectious disease research. Analysis of keywords and impactful publications identified three distinct developmental phases in this area that were significantly influenced by outbreaks of Zika and SARS-CoV-2 viruses. The research also highlights the synergistic efforts between academia and publishers in tackling global pandemic challenges. Through mostly consolidating research efforts, organoids are proving to be a promising tool in infectious disease research for both human and animal infectious disease. Their integration into the field necessitates methodological refinements for better physiological emulation and the establishment of extensive organoid biobanks. These improvements are crucial for fully harnessing the potential of organoids in understanding infectious diseases and advancing the development of targeted treatments and vaccines.

**Supplementary Information:**

The online version contains supplementary material available at 10.1186/s13567-024-01333-7.

## Introduction

Animal infectious diseases, resulting from pathogens such as viruses, bacteria, fungi, and parasites, significantly impact both animal health and productivity [1]. These diseases pose a zoonosis risk, where infections can cross species barriers, potentially leading to public health crises. Indeed, zoonotic infections can be transmitted from animals to humans, often through contaminated food, raising serious food safety and public health concerns. A notable example is *Salmonella*, a bacterium that causes an asymptomatic carrier state in poultry, yet causes 1.3 billion cases of gastroenteritis per year in humans [2]. Therefore, this not only leads to animal welfare issues and economic losses in agriculture but also poses human health risks to farm workers, visitors, and consumers of animal products. The threat extends to diseases in wildlife, as demonstrated by the Zika virus. Initially identified in a Rhesus macaque in 1947, Zika garnered global attention during the 2016 outbreaks, underscoring the broader public health implications [3]. Given the rising challenges of antibiotic resistance, pandemics, and nosocomial infections, research on animal and human infectious diseases remains crucial for the understanding and mitigating these threats.

The study of infectious diseases has traditionally relied on in vitro immortalized cell lines and in vivo animal models, each with significant limitations [4]. Cell lines, while maintaining consistent genetic profiles, often fail to replicate the complex characteristics of actual tissues, such as the architecture containing the different cell types of the tissue. In addition, some animal species remain without established cell lines, limiting the scope of in vitro research in these specific biological contexts. These limitations become particularly evident with pathogens such as noroviruses, which cannot replicate in cell lines. Consequently, much of our understanding of human norovirus (HuNoV) infections has been derived from clinical trials involving human subjects in the absence of a reliable in vitro model that accurately mirrors the virus' behavior [5]. This gap has hindered a comprehensive understanding of the HuNoV life cycle, its stability, antigenic diversity, and evolutionary dynamics. In contrast, while animal models offer more comprehensive systems for studying animal-specific infectious diseases, they often do not accurately represent human disease mechanisms due to physiological differences between species. Ethical considerations in animal experimentation have also grown, leading to the establishment of the 3R principle (Replacement, Reduction, and Refinement) by Russell and Burch in 1959 [6]. These ethical imperatives have underscored the need for alternative research models that not only more closely reflect human pathophysiological conditions but also adhere to higher ethical standards.

Recently, the advent of organoids, self-organized three-dimensional cell cultures, has marked a significant leap forward [7]. Derived from embryonic, induced pluripotent, or adult stem cells, organoids faithfully replicate the structure and function of the tissue they represent. In infectious disease research, organoids have become particularly invaluable by recapitulating many characteristics of in vivo disease and providing new insights into studies of host‒pathogen interactions [4]. They also open new possibilities for testing therapeutic interventions. This shift toward organoid-based research methodologies represents a key advancement in the field, enhancing our capacity to dissect the complexities of infectious diseases and fostering the development of more targeted and effective health care solutions.

Bibliometric analysis serves as a vital quantitative tool, enabling the assessment of research dissemination and evolution within specific research fields [8]. This methodology, by analyzing factors such as publication trends, citation counts, collaborative networks, and keyword prevalence, provides insightful perspectives on the development and influence of scientific contributions. Particularly in the biomedical research sector, bibliometric analysis plays a crucial role in pinpointing emerging research areas, evaluating the impact of publications, and guiding research policy decisions [9]. Despite its significance, there has been a notable absence of bibliometric analysis on the use of organoids for modeling infectious diseases. Such an analysis is imperative to quantitatively assess the growth of infectious disease research thanks to the development of organoid models.

The aim of this study was to conduct an analysis on the application of organoids for modeling human and animal infectious diseases. The animal, in this context, encompasses various categories including farm animals (e.g., pig, cow, chicken), domestic animals (e.g., dog, cat), wild animals (e.g., bat, chimpanzee), and experimental animals (e.g., mouse). This analysis allowed us to identify key publications, leading authors, and prominent institutions, as well as tracing the evolution of central research themes and uncovering emerging trends. The ultimate goal is to provide a clear understanding of the current landscape and future directions of infectious disease research facilitated by organoid models, identifying potential avenues and prospects within the existing body of literature.

## Materials and methods

### Data collection and retrieval strategies

Data were collected from the Web of Science Core Collection (WoSCC) on October 10, 2023. The search query used was “(((((((((ALL = (infection)) OR ALL = (infestation)) OR ALL = (infections)) OR ALL = (infestations)) OR ((((ALL = (fungi)) OR ALL = (fungus)) OR ALL = (mold)) OR ALL = (molds))) OR ((ALL = (bacteria)) OR ALL = (eubacteria))) OR ((ALL = (viruses)) OR ALL = (virus))) OR ((ALL = (parasites)) OR ALL = (parasite))) OR (ALL = (pathogen))) AND ((ALL = (organoids)) OR ALL = (organoid))”. According to the contemporary criteria for organoids, the first organoid was established in 2009. Therefore, we included publications spanning from 2009 to 2023. Our data extraction was confined to articles and reviews published in English and indexed in databases such as the Science Citation Index Expanded and Social Sciences Citation Index. The CD5 index is computed using citations up to 5 years in the future [10]. Hence, we selected research studies published before 2019. We then used the OpenAlex database to query which papers cited or were cited by those papers of interest.

### Data analysis

Every article obtained underwent manual selection to ensure its alignment with the established criteria. Details such as the type of organoid utilized, the pathogen involved, and the resources employed were individually recorded. The selected publications were then imported into bibliographic management software, where an exhaustive characterization was performed. This included the extraction of indexes such as the date of publication, document type, contributing authors and affiliated institutions, geographic origin, and keyword descriptors. Trend analyses were conducted to identify fluctuations in scientific contributions across time frames, the aging of references, the productivity and collaborative networks of authors and institutions, citation metrics, and keyword clustering, evolution, and bursts in citation frequency. The bibliometric assessments and network visualizations were executed using VOSviewer version 1.6.19 and CiteSpace version 6.2. R1 (64-bit). OpenAlex was accessed through its API using the “pyalex” Python module [11]. With the API, we queried information about all the papers of interest, then all the papers that they cite, and finally all the papers that cite them. With these data we could build the graph with papers as vertices and edges representing citing relationship (e.g. “paper A cites paper B”). Finally, the CD5 index was computed, for each paper of interest, using this graph and the “cdindex” Python module [10]. The script to perform the data collection from OpenAlex is available as additional file 1.

## Results

### Publication trends

A total of 609 publications were retrieved from the WoSCC, comprising 435 articles and 174 reviews. These works were published in 233 distinct journals, authored by a collective of 4198 researchers from 1159 institutions, and spanned 60 countries. Over a decade since its inception, the field of organoid-based research remains a rapidly evolving landscape. According to the Price Index [12], this area shows a high rate of innovation, with a score of 87.7%, which is faster than that observed in the fields of physics and biology (60–70%).

$$Price^{\prime}s\,index\, = \,\frac{number\,of\,citations\,less\,than\,5\,years\,old}{{total\,number\,of\,citations}}\, \times \,100\%$$

**Figure 1:**
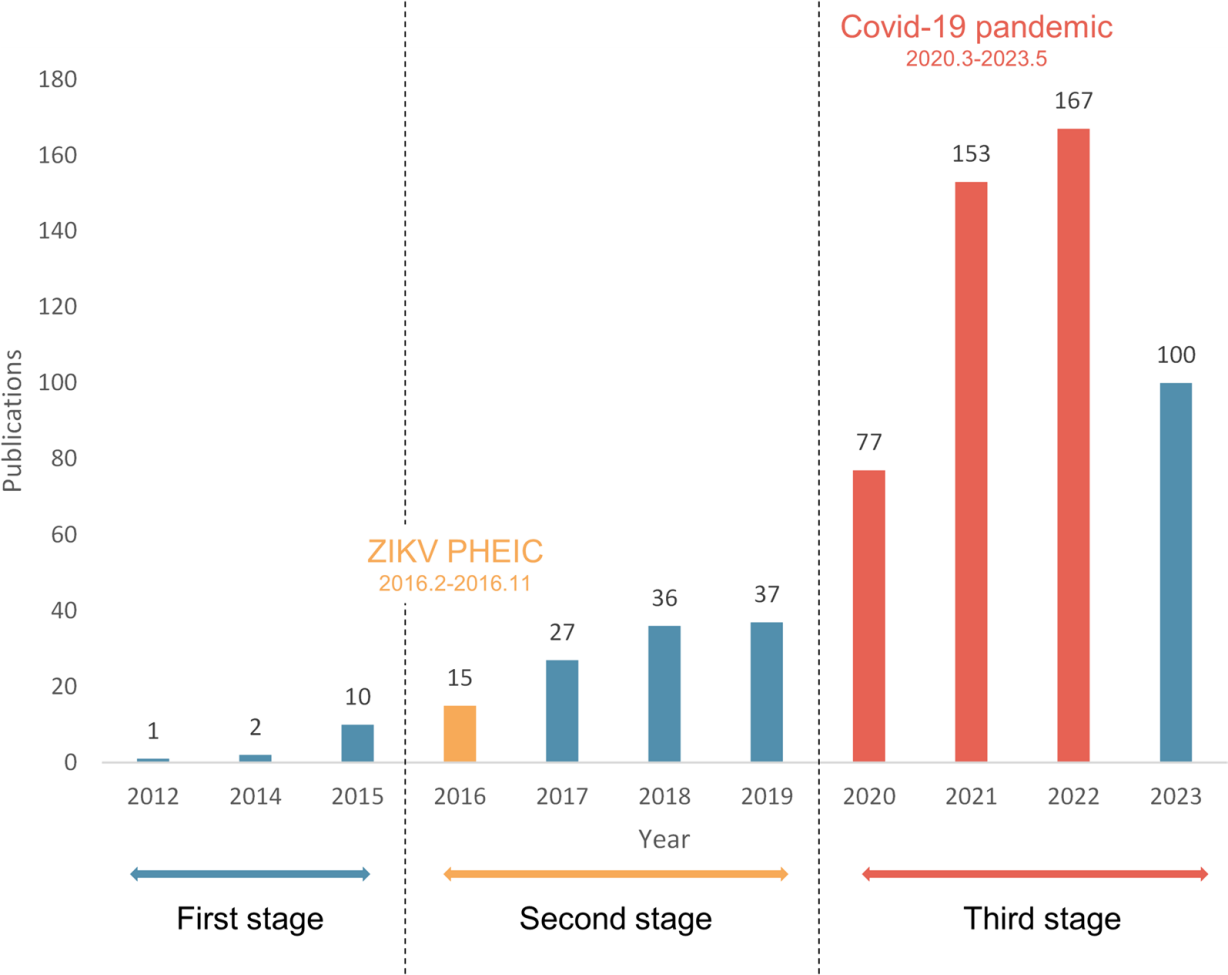
**The publication trends and important events of infection research on organoids.** Based on 609 publications collected between 2009 and October 10, 2023 (see Materials and methods for search query). *ZIKV* Zika virus, *PHEIC* Public Health Emergency of International Concern, *COVID-19* Coronavirus disease 2019.

Additionally, the cited half-life is the median age of the articles that were cited in the Journal Citation Report (JCR) year [13]. The cited half-life of publications in this field is approximately 2 years, indicating a quick renewal of scientific contributions. Research involving the use of organoid models for modeling infectious diseases has witnessed a substantial increase since the first study in 2012 [14]. This growing of literature can be categorized into three distinct stages, as illustrated in Figure 1. The initial stage, spanning from 2012 to 2015, represents an “early exploration” period where the number of publications did not exceed 10 papers per year. The second stage, from 2016 to 2019, marks a significant increase in the rate of publication, ranging from 10 to 40 papers per year. The third stage is that of accelerated growth, with annual publications soaring to a range of 70–167 papers.

**Figure 2:**
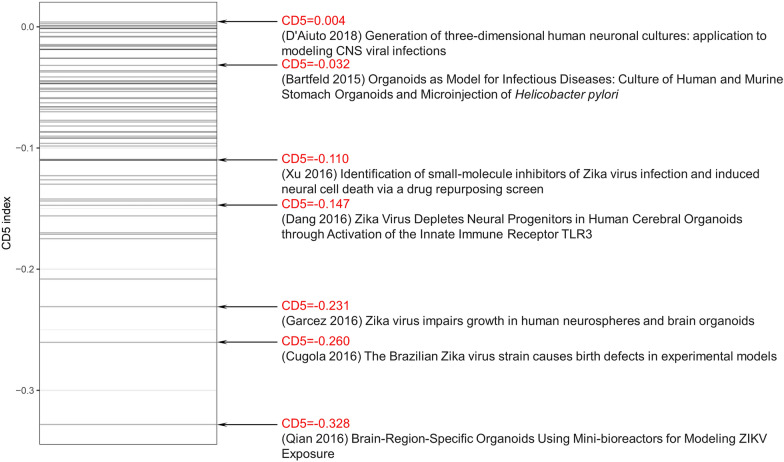
**CD5 index of infection research on organoids for the period 2012–2018.** The y-axis displays the CD5 index, with each line representing a specific publication. CD5 index values for the most disruptive, the most consolidating research, and those high-impact studies published before 2019 are shown on the right.

From the pool of 609 publications, 65 selected research studies published from the period 2012 to 2018 were identified as publications of interest for the CD5 index analysis. In accordance with the methodology introduced by Park et al. [10], we utilize the CD index to delineate the consolidating or disruptive attributes of scientific and technological endeavors. The rationale behind this lies in the conceptual framework where a disruptive element denotes a publication or patent that introduces novel concepts or ideas divergent from existing paradigms, thereby instigating transformative impacts within its respective domain. Subsequent research referencing a disruptive work typically exhibits diminished citation continuity with its predecessors, indicative of the pioneering essence of the contribution. Conversely, a consolidating element builds upon established knowledge, incrementally expanding the existing framework while maintaining continuity with prior endeavors. These contributions serve to reaffirm the significance of antecedent research, fostering cumulative progression within the field. By quantifying these distinctions, the CD index emerges as a valuable instrument for discerning the consolidating or disruptive nature inherent in scientific and technological progressions, thereby facilitating nuanced analyses of knowledge dissemination and innovation dynamics within research ecosystems. Specifically, we assessed the CD index 5 years after the publication year of each paper, denoted as CD5. According to our calculations, the average CD5 for the selected publications of interest is −0.08 (Figure 2). This indicates a slightly consolidating or cohesive citation pattern, although it is close to neutral. On average, the cited references in these publications do not strongly lean towards consolidation or disruption, suggesting a moderate level of cohesiveness in the scholarly discourse surrounding the organoid technique for studying infectious diseases.

**Figure 3:**
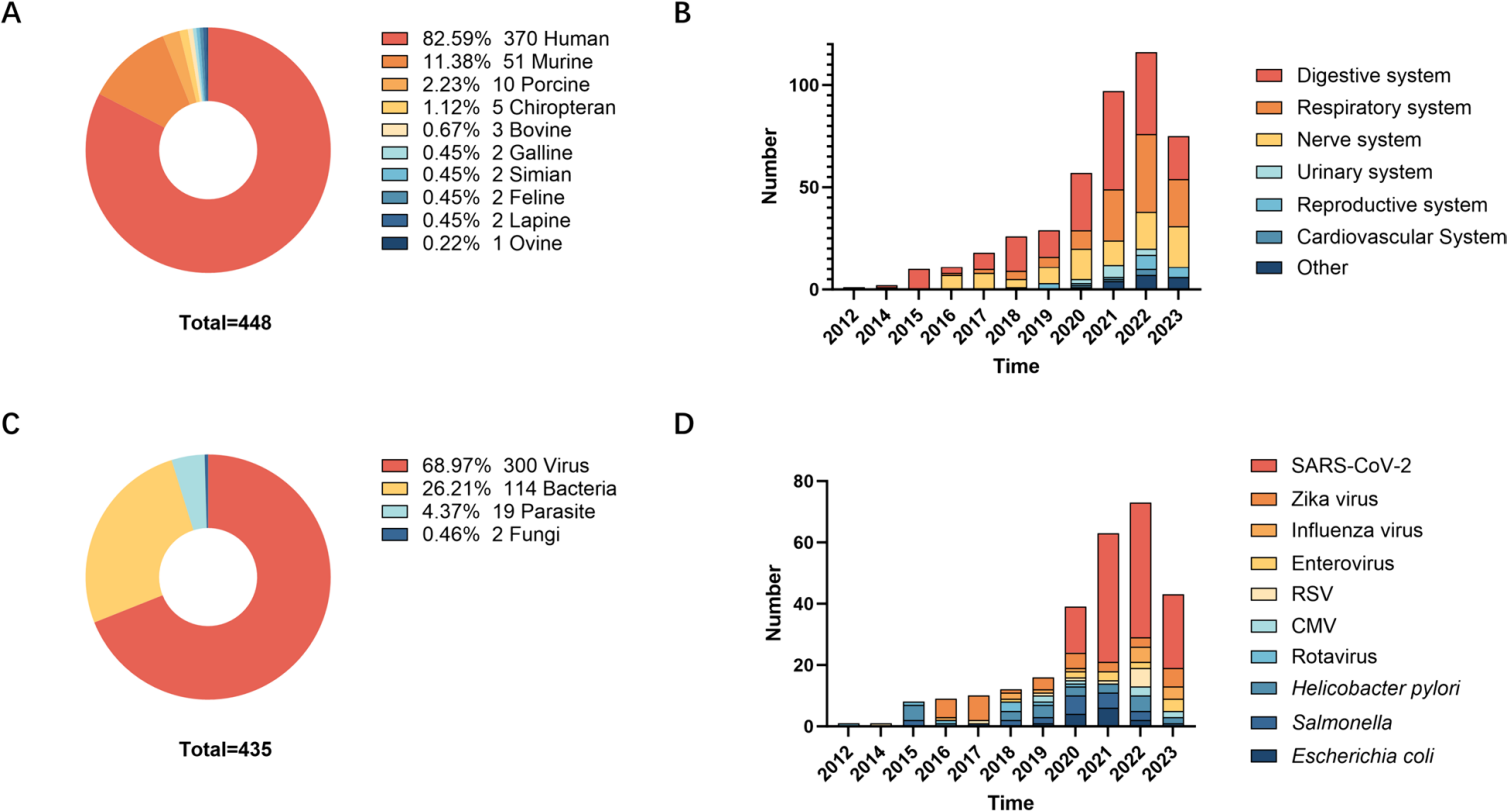
**Distribution of the number of articles for the period 2009–2023. A** According to each species group **B** per year according to tissue source **C** according to each pathogen group **D** per year according to the top 10 pathogen groups. (The total number of cases is different from the number of articles because more than one species was included in some publications). RSV: respiratory syncytial virus, CMV: cytomegalovirus.

When examining the sources of organoids, the majority were derived from human or murine tissues, as shown in Figure 3A. Other models are primarily utilized in animal husbandry research (Additional file 2). For the systems represented by these organoids, the digestive system—and particularly the intestinal organoids—continues to dominate as the primary model for studying infectious diseases (Figure 3B). Viruses are the most frequently studied pathogens (Figure 3C). In alignment with these epidemics, the bulk of the research on ZIKV (*n* = 36) and SARS-CoV-2 (*n* = 125) coincides with the timing of their respective outbreaks (Figure 3D). They are then followed by studies on *Helicobacter pylori* [15], *Salmonella* [16], *Escherichia coli* [17], and mycobacteria [18], which continue to progress steadily, while those on mycobacteria are just emerging.

### Author and institution analysis

**Figure 4:**
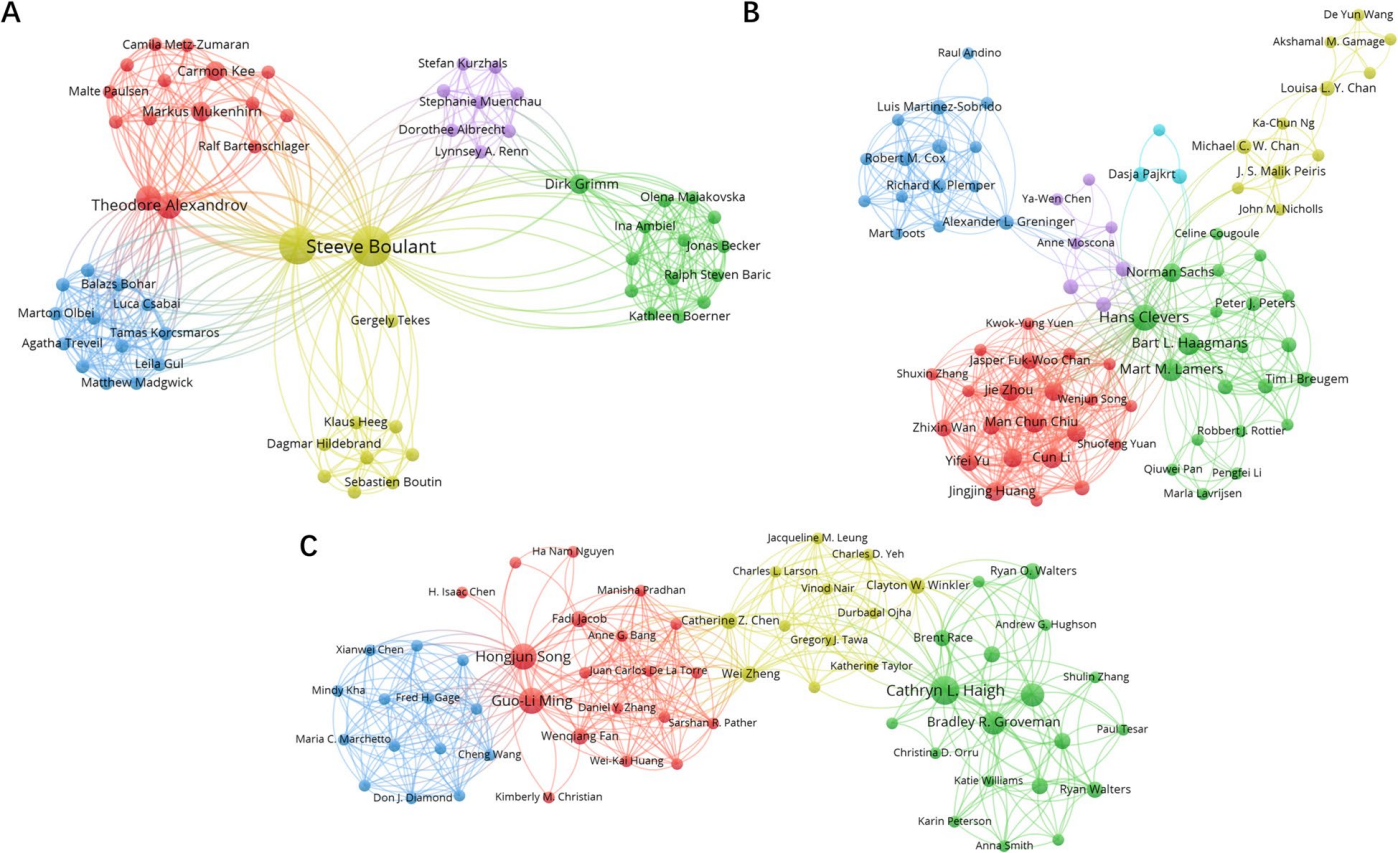
**Distribution of the author cooperation network for the period 2009–2023.** According to **A** intestinal organoid, **B** airway organoid, and **C** cerebral organoid. Colored clusters depict groups of collaborating authors (the colors used in the figure simply serve to differentiate between clusters, without implying any direct correlation with pathogen or animal species). The size of each circle corresponds to the number of publications, with larger circles indicating a higher publication count. Connecting lines signify collaborations between authors, with thicker lines signifying more frequent collaborations.

According to Price’s Law, half of the total research output in a given field comes from the square root of the number of researchers publishing in that field. It defines the minimum number of publications for core authors in a field: $m=0.749\times \sqrt{{n}_{max}}$ (${n}_{max}=22$ according to VOSviewer). Therefore, 101 authors with more than 4 publications in this area are defined as “main authors”. In Figure 4, we visualized the author cooperation network across the three primary organoid types: intestinal, airway, and cerebral organoid. The depicted network reveals relatively weak collaboration within different subgroups, indicating limited cooperation among authors in these specific areas. This observation suggests that there is room for enhanced collaborations and knowledge exchanges within the subfields of intestinal, airway, and cerebral organoid for research into the connections between these organs during infection.

The top 10 high-impact authors in this specialized field are listed in Table 1. Authors from the Netherlands unambiguously hold a leading role. Notably, researchers from Erasmus Medical Center often coauthor papers, thereby sharing their publications and citations. Other distinguished contributors to the field include Ali Mirazimi from Sweden, Jens Puschhof from Germany, and Kenneth Kak-yuen Wong from China, each of whom has made significant independent contributions.

**Table 1:** **High-impact authors**.

Rank	Author	Publications	Citations	Citation/publication	Institution	Country
1	Peters, Peter J	6	1881	313.5	Maastricht University	Netherlands
2	Mirazimi, Ali	5	1552	310.4	Unit of Clinical Microbiology	Sweden
3	Mykytyn, Anna Z	5	1302	260.4	Erasmus Medical Center	Netherlands
4	Riesebosch, Samra	5	1302	260.4	Erasmus Medical Center	Netherlands
5	Schipper, Debby	5	1302	260.4	Erasmus Medical Center	Netherlands
6	Puschhof, Jens	5	1169	233.8	German Cancer Research Center	Germany
7	Haagmans, Bart L	10	1516	151.6	Erasmus Medical Center	Netherlands
8	Lamers, Mart M	10	1516	151.6	Erasmus Medical Center	Netherlands
9	Clevers, Hans	22	2627	119.4	Hubrecht Institute	Netherlands
10	Wong, Kenneth Kak-yuen	5	549	109.8	The University of Hong Kong	China

**Figure 5:**
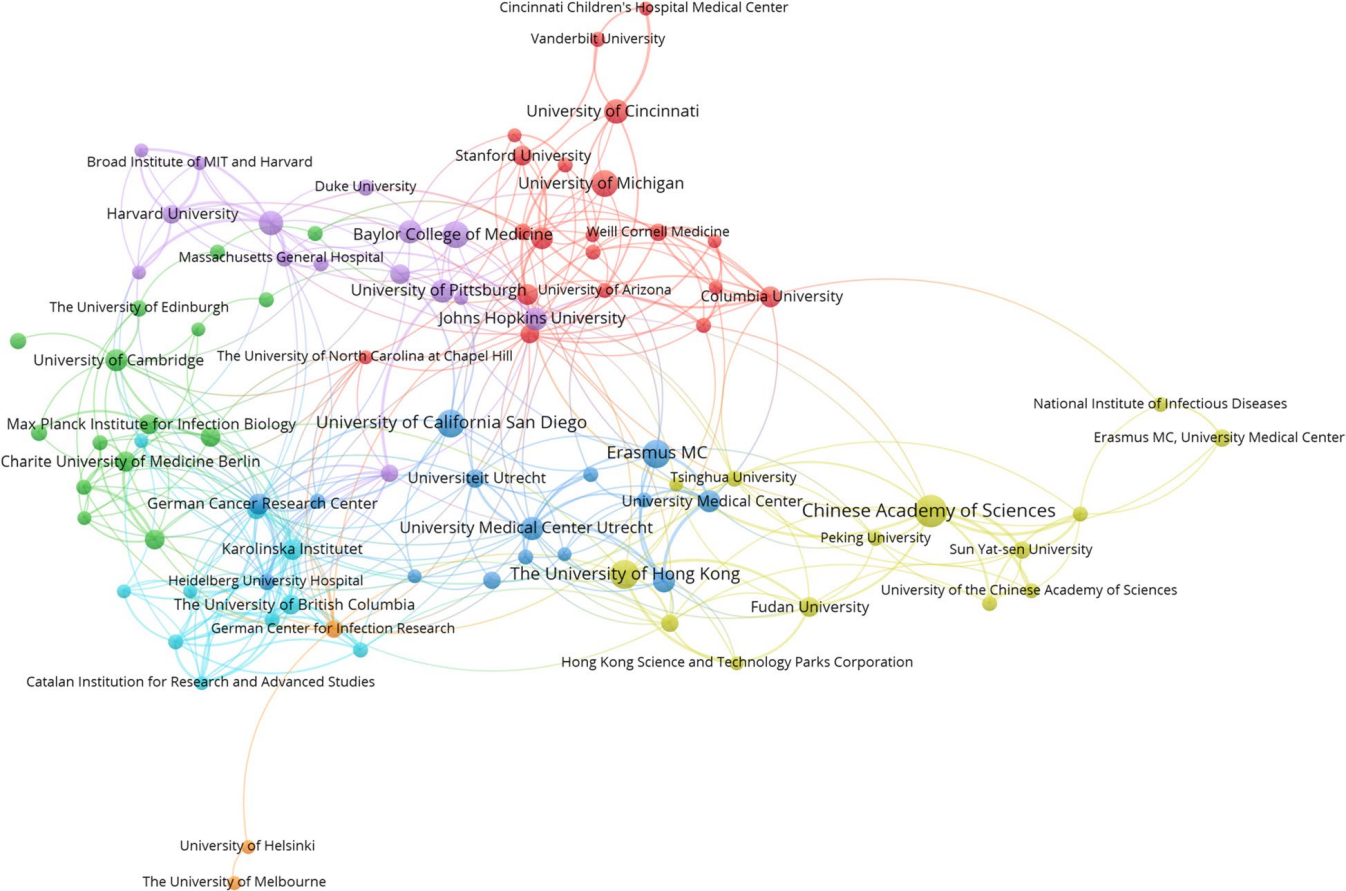
**Distribution of the institution cooperation network for the period 2009–2023.** Colored clusters depict groups of collaborating institutions. The size of each circle corresponds to the number of publications, with larger circles indicating a higher publication count. Connecting lines signify collaborations between institutions, with thicker lines signifying more frequent collaborations.

The top 10 high-impact institutions contributing to this field are outlined in Table 2. In contrast to the author clusters, these institutions are geographically dispersed across multiple countries. Their collaborative networks are both free-form and complex, transcending national borders, as shown in Figure 5.

**Table 2:** **High-impact organizations**.

Rank	Organization	Publications	Citations	Citation/publication
1	Maastricht University	6	1842	307.0
2	Catalan Institution for Research and Advanced Studies (ICREA)	5	1517	303.4
3	The University of Texas Medical Branch (UTMB)	5	1478	295.6
4	Karolinska University Hospital	5	1474	294.8
5	National Veterinary Institute	6	1574	262.3
6	Austrian Academy of Sciences	6	1560	260.0
7	University of Toronto	6	1556	259.3
8	Universidade Federal do Rio de Janeiro	5	1021	204.2
9	Emory University	11	2190	199.1
10	Institut Pasteur	5	987	197.4

### Journal analysis

The top 10 journals with the highest contributions to this field are enumerated in Table 3. Remarkably, nine out of these ten journals belong to Q1 in the JCR and possess high impact factors (IF). General journals such as *Nature*, *The EMBO Journal*, *Cell*, *Journal of Experimental Medicine*, *Cell Reports*, and *Science Advances* are represented, reflecting the interdisciplinary appeal of organoid research. In contrast, specialized journals such as *Gastroenterology* focus on gastroenterological research, *Cell Stem Cell* is dedicated to stem cell analysis, and *Infection and Immunity* as well as *Cell Host & Microbe* specifically concentrate on pathogen infection and immune responses.

**Table 3:** **High-impact journals (JCR and IF 2022–2023)**.

Rank	Journal	Publications	Citations	Citation/publication	JCR section	IF
1	Nature	7	2247	321.0	Q1	64.5
2	The EMBO journal	5	999	199.8	Q1	11.4
3	Gastroenterology	6	1048	174.7	Q1	29.4
4	Cell	19	3117	164.1	Q1	64.5
5	Cell stem cell	19	2991	157.4	Q1	23.9
6	Journal of Experimental Medicine (JEM)	5	751	150.2	Q1	15.3
7	Cell reports	8	715	89.4	Q1	8.8
8	Infection and immunity	8	529	66.1	Q3	3.1
9	Cell host microbe	5	306	61.2	Q1	30.3
10	Science advances	5	275	55.0	Q1	13.6

JCR: Journal Citation Report, IF: impact factor.

### High-impact publications

The ten most-cited papers in the realm of infectious disease research employing organoid models are shown in Table 4. Predominantly, these papers are research articles published in top rank, Q1 journals with IF above 10. Three of these articles, all published in 2020, focus on the role of angiotensin converting enzyme 2 (ACE2) in SARS-CoV-2 infections in human airway, intestinal and kidney organoids. Meanwhile, half of the list is about research on ZIKV infections in cerebral organoids, all of which were published in 2016. An exception is the article “*Organoids as Model for Infectious Diseases: Culture of Human and Murine Stomach Organoids and Microinjection of Helicobacter pylori*". Despite its publication in a Q2 journal with an IF of only 1.113, it has amassed an impressive 486 citations since its 2015 release, largely due to its innovative microinjection technique for bacterial infection in 3D organoids [19]. Furthermore, the paper by Sachs et al., published in February 2019, was seminal in establishing the first long-term, pseudostratified airway epithelium model using adult human tissues. Infection with respiratory syncytial virus (RSV) recapitulates central disease features in this model [20]. This work has also received substantial citation and contributed significantly to subsequent research, including but not limited to, studies on SARS-CoV-2.

**Table 4:** **High-impact publications (JCR and IF at the year of publication)**.

Rank	Title	Article type	Author & year	Journal	JCR	IF	Citations
1	Inhibition of SARS-CoV-2 infections in engineered human tissues using clinical-grade soluble human ACE2	Research	Monteil et al. 2020 [54]	Cell	Q1	41.584	1410
2	Brain-region-specific organoids using mini-bioreactors for modeling ZIKV exposure	Research	Qian et al. 2016 [40]	Cell	Q1	30.410	1242
3	SARS-CoV-2 productively infects human gut enterocytes	Research	Lamers et al. 2020 [55]	Science	Q1	47.728	1029
4	The Brazilian Zika virus strain causes birth defects in experimental models	Research	Cugola et al. 2016 [38]	Nature	Q1	40.134	877
5	Zika virus impairs growth in human neurospheres and brain organoids	Research	Garcez et al. 2016 [39]	Science	Q1	37.205	798
6	Zika virus depletes neural progenitors in human cerebral organoids through activation of the innate immune receptor TLR3	Research	Dang et al. 2016 [42]	Cell stem cell	Q1	23.394	495
7	Neuroinvasion of SARS-CoV-2 in human and mouse brain	Research	Song et al. 2021 [56]	Journal of experimental medicine	Q1	17.579	487
8	Organoids as model for infectious diseases: culture of human and murine stomach organoids and microinjection of *Helicobacter pylori*	Research	Bartfeld et al. 2015 [15]	Journal of visualized experiments	Q2	1.113	486
9	Identification of small-molecule inhibitors of Zika virus infection and induced neural cell death via a drug repurposing screen	Research	Xu et al. 2016 [43]	Nature medicine	Q1	29.886	472
10	Long-term expanding human airway organoids for disease modeling	Research	Sachs et al. 2019 [20]	The EMBO journal	Q1	9.889	447

JCR: Journal Citation Report, IF: impact factor.

### Keywords analysis

**Figure 6:**
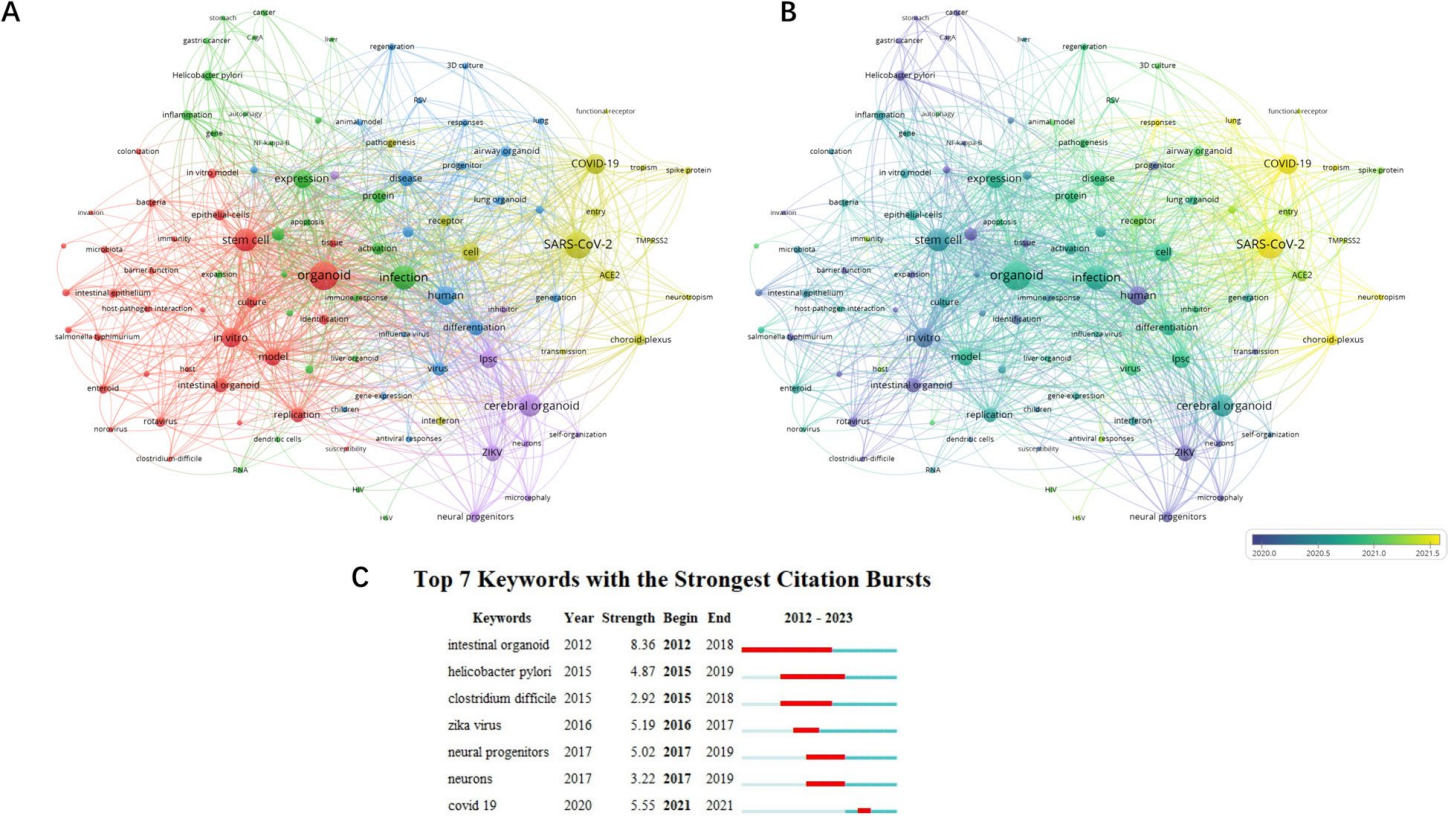
**Distribution of the keywords cluster, evolution, and citation bursts for the period 2009–2023.** The size of each circle corresponds to the number of publications, with larger circles indicating a higher publication count. Connecting lines signify co-occurrence between keywords, with thicker lines signifying more frequent co-occurrence. **A** keywords cluster: different colors represent different clusters; **B** evolution: the color represents the average publication year of each keyword; **C** citation bursts: “year” represents the first time the keywords appeared. “Begin” and “End” indicate the start and termination points of the period of heightened research activity on a specific keyword. “Strength” refers to the intensity of the burst during this period.

The top 100 cited keywords were categorized into five distinct clusters, as illustrated in Figure 6A. The largest cluster (red cluster) encompasses research on intestinal organoids and pathogens that commonly infect the intestinal tract, such as *Salmonella*, *Escherichia coli*, and rotaviruses. The second cluster (green cluster) focuses on *Helicobacter pylori* infection in gastric organoids and its mechanistic link to gastric cancer, as well as hepatitis B and C virus infection in liver organoids. The blue cluster is about using airway organoids to investigate influenza and RSV, while the yellow cluster employs airway organoids to study SARS-CoV-2, with particular emphasis on ACE2 and spike protein interactions. The smallest cluster (purple cluster) applied cerebral organoids to explore ZIKV infections.

The evolution of these keywords over time is mapped in Figure 6B. Research on gastrointestinal organoids to mimic infections involving pathogens such as *Salmonella*, *Clostridium difficile*, *Helicobacter pylori*, and rotaviruses—as well as research on ZIKV infection in cerebral organoids—features the earliest average publication years, attesting to their longstanding research history. Studies on influenza and RSV in airway organoids follow chronologically, whereas SARS-CoV-2 research is a rapidly emerging field in the context of the COVID-19 pandemic.

Figure 6C identifies the seven most cited keywords experiencing a citation burst, which aligns with the three phases previously discussed. The keyword “intestinal organoid” initiated the first and most significant burst, closely followed by two specific gastric intestinal pathogens: “*Helicobacter pylori*” and “*Clostridium difficile*”. ZIKV has instigated a surge in research involving cerebral organoids, which primarily consist of neural progenitors and their derived neurons. As of now, the keyword “COVID-19”, which started bursting in 2021, has only persisted for 1 year but already exhibits the second-highest burst strength.

## Discussion

The use of organoids in infectious disease research represents a significant paradigm shift, offering a more physiologically relevant and ethically sound alternative to traditional cell cultures and animal models. Our analysis reveals their potential in elucidating complex host‒pathogen interactions, particularly in the context of emerging infectious diseases. This approach not only enhances our understanding of disease mechanisms at the cellular and molecular levels but also opens new avenues for the development of targeted therapeutics and vaccines.

### The first stage: early exploration from 2012 to 2015

**Figure 7:**
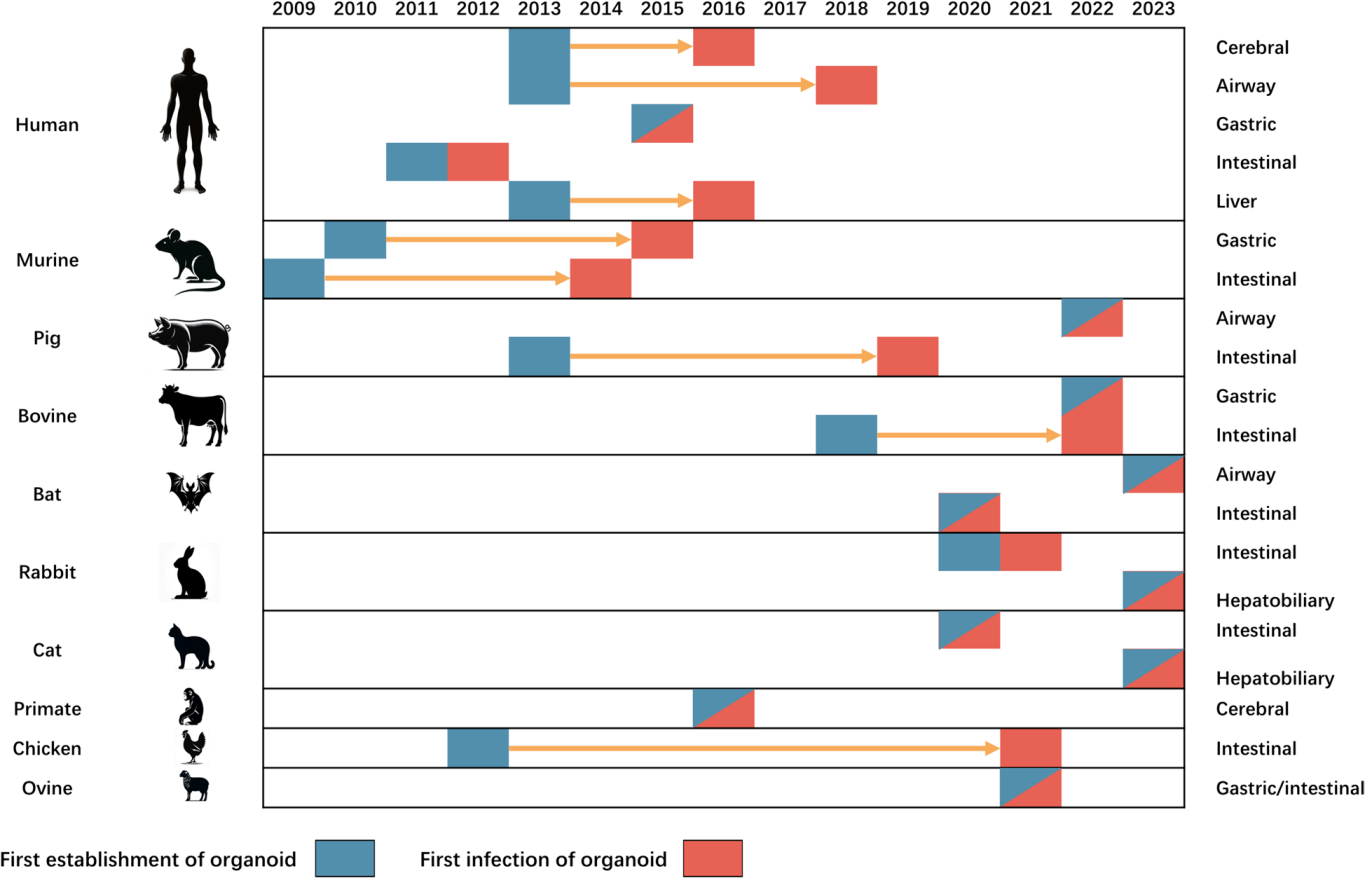
**Timeline of organoid development and initial infection across species and organs from 2009 to 2023**. The horizontal bars represent the timeline. The left y-axis lists the species: human, murine (mouse), pig, bovine (cow), bat, rabbit, cat, primate, chicken, ovine (sheep). The right y-axis categorizes the tissue sources involved in the research, including cerebral, airway, gastric, intestinal, and liver organoids. The blue segments mark the first establishment of the organoid model, while the red segments denote the first recorded study on host‒pathogen interactions using the organoid model. Arrows indicate the progression of research within each category. This visual summary underscores the advancements and applications of organoid models in infectious disease research over the last fourteen years.

In 2009, the Hans Clevers’ group pioneered the development of the first organoid derived from murine intestines, inaugurating a novel domain of research in gastrointestinal diseases [21] (Figure 7). An inaugural study utilizing an organoid model for infectious disease research emerged in 2012, elucidating the interaction of human intestinal organoids with rotaviruses by physically cutting open the organoid to allow virus access to epithelial cells [14]. This foundational work catalyzed subsequent research, including studies on *Helicobacter pylori* infections using gastric organoids [19], as well as research on human and murine intestinal organoids infected with *Salmonella* [16] and *Clostridium difficile* [22]. These initial investigations established the cornerstone for the expanding research domain of intestinal organoid infections, which emerged as the most prominent keyword cluster (depicted in red) in our analysis (Figure 6A). This cluster, along with investigations into gastric organoids infected with *Helicobacter pylori*, features the earliest average publication year, highlighting its sustained significance in the field (Figure 6B). A citation burst analysis delineates the “intestinal organoid” as exhibiting the highest burst strength, signifying its critical role in the initial phase of the field (Figure 6C). This is further augmented by frequent studies on two bacterial pathogens, “*Helicobacter pylori*” and “*Clostridium difficile*”, underscoring the seminal importance of these areas in early research.

Nevertheless, the aforementioned research encounters a significant challenge [23]. Although physiologically relevant, these organoids display polarized epithelial cells with a basolateral side facing outward and an apical side enclosed internally. Consequently, when bacteria approach these organoids externally, they interact with the basolateral surface, in contrast to in vivo conditions where the epithelial cells’ apical surface engages with luminal components, including microorganisms. This arrangement complicates direct access to the apical side of the epithelial cells in organoid models.

To surmount this challenge, researchers have developed and employed various methodologies. In 2014, researchers first cultivated murine organoid epithelial cells as 2D monolayers [24], a technique applied to pig [25], bat [26], rabbit [27], and bovine [28] intestinal organoid infection. This method involves seeding dissociated organoid cells on surfaces precoated with diluted extracellular matrix components such as Matrigel^™^. This format promotes the formation of a cohesive epithelial barrier, as evidenced by increasing transepithelial electrical resistance measurements, indicative of barrier integrity. The apical surface in these monolayers is readily accessible for experimental interventions with microorganisms, with differentiation induced either by niche factor withdrawal or by establishing an air–liquid interface. However, while 2D monolayers provide easier access to the apical surface, they lack the complex three-dimensional architecture intrinsic to intestinal organoids, such as crypt-like structures, which are essential for mimicking the in vivo cellular organization of the intestine. Subsequently, the microinjection technique introduces microorganisms directly into the luminal space of 3D organoids at the end of 2014, thereby facilitating interaction with the apical epithelium [15]. This novel technique was applied in 7 of 10 publications in 2015 to study bacterial infection of gastrointestinal organoids. It has also catalyzed research on diverse pathogens, including *Salmonella* [29], *Escherichia coli* [17], and mycobacteria [30]. Despite its ingenuity, this method requires specific equipment and is labor intensive. The microinjection in livestock intestinal organoids has also been reported by Beaumont et al. [31]. However, variability in injection volume and potential leakage into the extracellular medium challenge its reproducibility.

Additionally, the infection of disassociated organoids was also reported in 2015 to study transformation induced by *Salmonella* in gallbladder organoids [32], and further for the long-term model of *Chlamydia trachomatis* infection of fallopian tube organoids in 2019 [33]. Yin et al. report the direct infection of rotavirus on intestinal 3D organoids and its treatment response to antiviral medications [34]. With the proliferation of novel methodologies for infecting organoids according to diverse research targets, the exploration of organoid models in infectious diseases enters the second stage.

### The second stage: ZIKV-related explosion and the evolution of organoid models from 2016 to 2019

First introduced in 2013, human cerebral organoids have served as research models for neural development [35], neurodegenerative disorders [36], and even patient-specific microcephaly models using induced pluripotent stem cells (iPSCs) [37]. However, their application in infectious disease research remained unexplored until 2016 (Figure 7). Reacting swiftly to the ZIKV outbreak in Brazil, Patricia Pestana Garcez and her team from the University of São Paulo submitted a groundbreaking manuscript in March 2015 [38], at the same time with Brazil’s official reporting of the outbreak. They were the first to investigate ZIKV-induced neural defects in cerebral organoids, offering invaluable insights into the challenges the ZIKV epidemic presented. In 2016, the outbreak led to a Public Health Emergency of International Concern (PHEIC) from February to November of that year. A group of high-impact articles has emerged in leading journals since then (Figure 1). Garcez et al. [39] have continued to refine models that mimicked ZIKV-induced neural defects (Table 4). Concurrently, Qian et al. [40] developed a forebrain-specific organoid model from iPSC using a miniaturized spinning bioreactor to study both African and Asian strains of ZIKV. The CD5 index of this study ranks the lowest among all included publications (Figure 2), indicating its significant consolidation within this research domain. By integrating established insights into ZIKV pathogenesis with cutting-edge organoid technology, this investigation makes incremental advancements while maintaining coherence with prior research efforts. Through its elucidation of ZIKV tropism and its effects on neural progenitor cells, alongside its demonstration of organoid efficacy in drug testing, the study reaffirms the importance of preceding research and contributes to the ongoing cumulative progress in the field. In contrast, the research on CX7 HCS technology for modeling HSV-1 infection and assessing antiviral therapies [41] attains the highest CD5 score, signifying its substantial disruptive potential within this field (Figure 2). The capability to swiftly and accurately capture confocal z-stacks from complex multilayer cellular structures streamlines the screening process, obviating labor-intensive manual procedures and minimizing potential variability. Furthermore, the platform’s rapid data analysis capabilities facilitate high-throughput screening, thus expediting the pace of drug discovery endeavors. Further deepening the field’s understanding, Dang et al. [42] elucidated the activation of the TLR3 signaling pathway by ZIKV and its resultant depletion of neural progenitor cells, which led to reduced organoid size. In a novel approach, Xu et al. [43] deployed an ultrahigh-throughput drug screening platform based on cerebral organoids and discovered that Emricasan, a pan-caspase inhibitor, could mitigate ZIKV-induced caspase-3 activation.

Research fervor surrounding ZIKV persisted until 2019, as evidenced by the burst in citations for terms such as “Zika”, “neural progenitor”, and “neurons” (Figure 6C). Although publication growth slowed down with the subsidence of the ZIKV epidemic, scholarly activity in this domain has not ceased. Recent studies continue to delve into the mechanisms underlying ZIKV-induced neurological defects [44] and potential treatment avenues [45]. Consequently, keywords associated with ZIKV form the smallest cluster in our analysis and a relatively aged average publication year, indicating its shifting priorities (Figure 6A).

Despite all the innovation in infection models in the first stage, an alternative approach tries to reverse the polarity of organoid cells by removing the extracellular matrix [46]. This technique has been established with human organoids [47] and then adapted to pig [48], bovine [28], and chicken [49] organoids. However, polarity inversion is not observed in all organoids. Another method involving the exposure of intestinal organoids to an inflammatory cocktail has been shown to invert human organoid polarity [50], although its application in altering epithelial polarity in farm animal organoids remains unexplored. Although the application of the organoid model has greatly affected the area of infectious disease research, the CD5 index shows that its disruptiveness is relatively neutral. The observed slightly consolidating or cohesive citation pattern in the publications of interest may indeed be attributed to the organoid model's reliance on the foundations established by the cell line culture technique. Recognizing that the application of the organoid model in studying infectious diseases spans only around a decade, and considering its current limitations, traditional techniques such as cell line cultures and animal experiments remain crucial for validating results derived from organoid experiments. Nevertheless, we anticipate that as the organoid technique continues to evolve, it will progressively assume a more prominent role in the field of infectious diseases. This advancement may potentially lead to a reduced reliance on experimental animals, underscoring the ongoing shift towards more sophisticated and ethically mindful methodologies in scientific research.

### The third stage: SARS-CoV-2-related explosion from 2020 to the present

The initial airway organoid model, derived from murine sources, was established in 2009 [51], followed by human airway organoids reported in 2013 [37] (Figure 7). Subsequent research on a human airway organoid model focused on various infectious agents, such as RSV [20] and influenza viruses [52], together forming a keyword cluster represented in blue (Figure 6A). However, an advancement occurred in 2019 with the development of the first long-term pseudostratified airway epithelium organoid model using adult human tissues [20]. This pivotal innovation set the stage for numerous subsequent investigations, particularly those centering on SARS-CoV-2 and other respiratory pathogens. After the World Health Organization (WHO) declared COVID-19 a pandemic in March 2020, a wave of high-impact studies emerged, leveraging human-derived organoids from various organ systems [53] (Figure 1). The paper by Monteil et al. [54] stands as the most cited in this context, accumulating 1410 citations thus far (Table 4). Their research validated that pre-existing human recombinant soluble ACE2 (hrsACE2) could inhibit SARS-CoV-2 infections in both human capillary and kidney organoids, which might be a potential treatment for COVID-19. Supporting these findings, Lamers et al. [55] confirmed that SARS-CoV-2 could infect human small intestinal organoids and demonstrated the effectiveness of hrsACE2 in preventing viral binding. Song et al. [56] explored SARS-CoV-2 infection in human cerebral organoids, shedding light on resultant metabolic alterations. Keywords from these more recent studies form a yellow keyword cluster, characterized by a notably recent average publication year.

While both are propelled by viral outbreaks, research into SARS-CoV-2 is notably more comprehensive than earlier studies on ZIKV. It goes beyond simply unraveling the mechanisms of infection to include vaccine and drug development. Given the species-specific nature of viral infections and the distinct differences in adaptive immunity between species, traditional animal models often prove inadequate. Many vaccine candidates that succeed in animal trials ultimately fail in humans [57]. Consequently, innovative, human-specific testing methodologies could expedite drug and vaccine validation processes. Although human organoids lack an immune system, they offer unique advantages for understanding host‒virus interactions and for assessing the efficacy and neutralizing capabilities of potential vaccines [58]. Traditional cell lines are limited in replicating the physiologically relevant dynamics of in vivo SARS-CoV-2 infection, positioning human organoids as promising platforms for COVID-19 drug discovery. Specifically, human lung organoids have been employed to evaluate the effectiveness of drugs targeting viral entry and replication [59]. This multifaceted approach facilitates a more direct translation from research to clinical application, offering prompt and effective interventions against the virus. Moreover, the broad impact of SARS-CoV-2 on various organ systems has been matched with a versatile range of well-established organoid models. Research has incorporated cerebral [60], gastrointestinal [56], liver [61], and kidney [62] organoids, highlighting the solid groundwork and expansive potential of organoid-based research.

### Organoid source and pathogen trends

In our analysis, it is evident that the human is the primary species for organoid generation (Figure 3A). This preference is grounded in several key reasons. First, human organoids accurately mirror the structure and function of human organs, circumventing potential discrepancies due to species-specific physiological and disease susceptibility differences [4]. Second, advancements in biobanking and the refinement of ethical consent processes have enhanced the availability of human sources for research, making them a practical option for organoid creation [63]. This approach also mitigates ethical concerns often linked with animal model usage. Furthermore, human organoids have demonstrated superior predictability in assessing the efficacy and toxicity of new therapeutic agents, offering critical insights into drug development [64].

Moreover, the majority of organoid research focuses on viruses, followed by bacteria (Figure 3C). This emphasis on viruses is attributed to several factors. Firstly, organoids facilitate the cultivation of otherwise uncultivable viruses. For instance, noroviruses, which cannot replicate in conventional cell lines, are capable of infecting human intestinal organoids [5]. Additionally, viruses can be directly amplified in organoid models from clinical isolates, negating the need for mutation or adaptation [65]. Secondly, the imperative created by recent significant epidemics has accelerated research in this area [53]. Similarly, organoids offer a promising approach to surmount current challenges of in vitro parasitic infections and to provide a mechanistic understanding of the impact of parasites on their host environments [66]. However, due to the complexity of parasite life cycles and the size of parasites such as gastrointestinal nematodes that are generally 10 to 10 000-fold larger than viruses, bacteria and protists, the studies using organoids as models of parasite-host interaction have been considerably delayed.

While human and murine models have been the mainstay of organoid research, their application in animal models is expanding rapidly. Figure 6 illustrates the time lag between the establishment of species-specific organoids and their application in infectious disease research. Human and murine organoids, largely developed before 2016, usually had a lag of approximately 5 years before being applied to infectious disease research. In contrast, most animal organoids, apart from pig [48] and chicken [67] intestinal types, were developed after 2016. The integration of pig and chicken organoids into infectious disease research took approximately 7 and 5 years, respectively. Recent years have marked a notable increase in the development of animal organoids, signifying rapid advances in the establishment of animal organoids and their utilization in infectious disease research. It will be comprehensively discussed in the following section.

### Farm animal, domestic animal, and wild animal organoids model in infectious disease research

Additional file 2 highlights 26 publications delving into infectious disease research using farm, domestic, and wild animal organoid models, offering unique systems to match host with pathogen tropism. Pig intestinal organoids stand as the most widely employed veterinary organoid model in current research. Out of the ten studies conducted, all but one focused on swine coronaviruses, particularly swine enteric coronaviruses (CoVs). This subgroup comprises porcine epidemic diarrheal virus (PEDV), transmissible gastroenteritis virus (TGEV), porcine deltacoronavirus (PDCoV), and swine acute diarrhea syndrome coronavirus (SADS-CoV). These viruses pose significant threats to piglets, causing widespread illness and mortality, resulting in substantial economic losses in major pig-producing nations due to endemics or large-scale epidemics [68]. Therefore, finding effective treatments for CoVs remains a challenge. Researchers often use non-porcine cells, which cannot fully mimic the real infection process due to their lack of interferon production [69]. Pig intestine epithelial cell lines, such as IPEC-J2, still struggle to replicate the complexity of in vivo epithelia [70]. The absence of a reliable in vitro system mimicking in vivo CoVs infection makes it tough to thoroughly study CoVs and develop effective strategies against it.

To address these challenges, pig intestinal organoids and virus infection models have been developed. Studies indicate successful virus infection, replication, and disruption within these organoids, faithfully recapitulating the in vivo PEDV infection process. Leveraging these models, researchers have delved into the effects and mechanisms of virus infection on epithelial proliferation [71] and barrier function [25]. Additionally, the protective effect of pig milk small extracellular vesicles (sEV) against PEDV was validated in the organoid model, aligning with observations from cell lines and in vivo experiments [72]. Furthermore, an extended pig airway organoid, capable of sustaining long-term cultures, was developed for the study of porcine respiratory coronavirus (PRCoV) [73]. Both 3D and 2D airway organoids proved permissive for PRCoV infection, establishing versatile platforms for studying respiratory coronavirus dynamics and test therapeutic strategies.

Similarly, gastrointestinal and hepatobiliary organoid models have been successfully established in bovine [66], ovine [74], rabbit [27], cat [27], and chicken [16] species, showcasing stable storage and long-term passage capabilities. Beyond various viruses, these models have been employed to study bacteria such as *Mycobacterium avium* subspecies *paratuberculosis* [28], and *Salmonella* [16], as well as parasites like gastrointestinal nematode [66], *Teladorsagia circumcincta* [74], and apicomplexan *Eimeria* [49]. These studies, all published after 2020, mark pioneering efforts in creating organoid models and investigating pathogen infections.

Wild animal organoids, distinct from those of farm animals, focus on investigating zoonoses suspected to originate from wildlife. The Zika virus (ZIKV) outbreak in Brazil (ZIKV^BR^) differed from its African counterpart (ZIKV^AF^), linked to African primates. Studying the association of ZIKV with microcephaly and birth defects, especially with ZIKV^BR^, is essential. Cerebral organoids from non-human primate pluripotent stem cells revealed different infection kinetics between ZIKV^BR^ and ZIKV^AF^ [38]. For SARS-CoV-2, bat organoids were developed, with *Rhinolophus sinicus* intestinal organoids supporting robust viral replication [75]. Higher interferon levels in these organoids may inhibit the inflammatory response to SARS-CoV-2 [76]. However, intestinal organoids from *Rousettus leschenaultii* [77] and airway air–liquid interface from *Eonycteris spelaea* [26] did not support SARS-CoV-2.

## Limitations and perspective

The research progress after the Zika virus (ZIKV) and SARS-CoV-2 outbreaks demonstrates a notably quicker and more comprehensive adoption of organoids in studies related to SARS-CoV-2, encompassing areas from fundamental mechanistic research to the development of vaccines and therapeutics. This rapid integration can largely be credited to the profound global impact of the SARS-CoV-2 pandemic and the pre-existing experience with airway organoids in various pathogenic investigations, including those involving viruses, bacteria, and other microbes [78]. This prior groundwork paved the way for the expedited adaptation and application of organoid infection models and methodologies in SARS-CoV-2 research.

Concurrently, it is vital to focus on developing more physiologically representative organoid models, particularly those incorporating the environment, such as mechanical force, microbiota, and immune cell co-cultures [79]. Such an advancement would not only improve the ability of these models to simulate physiological conditions but also deepen our understanding of the intricate interactions between pathogens and the host’s immune system. In the domain of animal research, the emphasis has predominantly been on organoids derived from stem cells or embryonic stem cells [80]. The exploration and utilization of iPSC are essential. iPSC can offer a wider array of organoid models, facilitating more detailed studies across different animal species and organ systems. This is critical for comprehending the variations in disease mechanisms and responses to treatments across species, which in turn would contribute significantly to the fields of comparative biology and translational medicine.

Moreover, the application of organoids should be expanded beyond just investigating infection mechanisms to encompass the areas of drug and vaccine development and screening [43]. The establishment of extensive organoid biobanks would be instrumental in accelerating the identification and selection of patient-specific therapeutic agents [63], thereby improving preparedness for a range of pathogens, potentially rising with global warming.

This study presents a bibliometric analysis focusing on the application of organoid models in human and animal infectious disease research. Our findings show a developmental trajectory, highlighting the growing importance of organoids in this field. This shift toward organoid models in medical and veterinary research represents a promising alternative to traditional methods, potentially reducing the reliance on animal testing. The study underscores the value of these models in enhancing our understanding of infectious diseases. In addition, it points toward a future where veterinary research can benefit from the scientific advancements offered by human organoid technology. In conclusion, the evolution of organoid models holds great promise for veterinary infectious disease research, offering a path toward more ethical and effective disease management in animals that can be transmitted to humans.

### Supplementary Information

**Additional file 1Script to compute CD5 index**. This file contains the script to perform the data collection from OpenAlex..

**Additional file 2** **Farm, domestic, and wild animal organoids in infectious disease research**. This file contains the table listing the studies on farm, domestic, and wild animal organoids in infectious disease research (IF at the year of publication).

## Data Availability

Additional data from this study can be obtained by contacting the corresponding author. Other information from this study can be obtained, upon reasonable requests, from the corresponding author.
